# Hotel Employees’ Burnout and Intention to Quit: The Role of Psychological Distress and Financial Well-Being in a Moderation Mediation Model

**DOI:** 10.3390/bs13020084

**Published:** 2023-01-19

**Authors:** Asier Baquero

**Affiliations:** Faculty of Economics and Business, Catholic University of Murcia (UCAM), 30107 Murcia, Spain; abaquero@ucam.edu

**Keywords:** workplace behavior, burnout, intention to quit, psychological distress, financial well-being, hotel industry, PLS-SEM

## Abstract

Continuous changes, such as pandemics and increasing competition, as well as high workload, affect the workplace behavior of hotel organizations today, resulting in employee burnout and intention to quit. The purpose of this research was to investigate the effect of burnout on intention to quit among male hotel employees, integrating the mediating effect of psychological distress and moderating effect of financial well-being. Male employees in four- and five-star hotels in the UAE completed a total of 304 questionnaires. All direct relationships were positive and statistically significant, there was a partial mediating relationship, and only one of the moderating effects was statistically significant. This study found that burnout predicts the intention to quit as well as psychological distress. Psychological distress partially mediates the relationship between burnout and the intention to quit. Financial well-being moderates the relationship between burnout and psychological distress—making this relationship stronger for employees with high-income prospects—but not the relationship between burnout and intention to quit; regardless of the financial well-being of the employee, burnout will lead to the intention to quit their job. Hotel organizations must be aware of the consequences of employee burnout and concentrate on identifying and treating its causes.

## 1. Introduction

The hotel industry has witnessed considerable changes over the years, with some issues, such as the COVID-19 pandemic exposing its employees to burnout (BU). BU is an emotional state arising from psychosocial weariness when one fails to cope with job-related stress [1]. Scholars maintain that the problem’s prevalence draws from BU syndrome, which, according to [2,3], often propels workers into job dissatisfaction, especially when they are facing changing work roles and expectations without support. Despite BU’s existence in several industries, the issue is prevalent in the hospitality/hotel industry globally, prompting employees to develop intentions to quit. Critical contributors to this emotional exhaustion involve excessive workload and long working hours for hotel employees. These factors expose the affected laborers to worry and anxiety if they cannot perform the assigned tasks within the set timelines [2,4,5]. The pressure may escalate into frustrations, particularly when “employees are forced to neglect some aspects of their jobs and life” [5] (p. 399). In return, hospitality workers may feel incapable of handling and matching their job demands, heightening their intention to leave. Since these experiences create BU, they depict a positive correlation between the latter and hotel workers’ intention to quit (ITQ).

Psychological distress (PD) arises from emotional suffering linked with demands and stressors that are challenging to cope with in one’s daily life. Individuals experience BU syndrome when they cannot handle workplace emotional stress, causing emotional exhaustion [6]. As a result, the sufferer develops a “vicious cycle of depersonalization and decreased personal achievement” [6] (p. 20). Since BU-contributing factors are multifactorial, the authors maintain that its persistence can increase one’s chances of developing job-related stress, leading to anxiety and depression when not controlled. Refs. [6,7] concur, referring to the latter impacts as PD. According to them, increased depression and anxiety are results of BU syndrome and can cause more deterioration in an individual’s physical and mental health and job performance. Ref. [8] links BU with propagated workplace bullying, insisting that encouraging vice can facilitate workers emotional exhaustion, lowering their mindfulness and resilience and exposing them to PD. Despite focusing on medical students, [9] admit that higher workloads can augment one’s experience of BU, subjecting them to PD. These explanations prove the existence of a direct relationship between BU and PD, which, unfortunately, limits a person’s performance.

PD’s prevalence depends on individuals’ relationships with their environments. Changing distressing or undesirable situations that cause this problem requires coping and appraisal [10]. However, the authors decry PD as propagated by high BU levels and fatigue, hindering one’s ability to overcome it. Ref. [11] links PD with reduced work satisfaction, which increases turnover intentions. Workers often feel distressed when subjected to intensified “low wages, professional invalidation, and limited career progression” [12] (p. 2). Other factors include abusive leadership, which [13] maintains often attenuates workers’ poor psychological well-being, contributing to greater turnover intentions. While some can cope and shift to finding ways that boost their morale to stay at their jobs, others view such work-related factors as unfavorable, prompting them to develop the urge to quit [14]. Employees hardly leave their jobs before engaging in problem management and emotional regulation as coping strategies [10]. They only choose to quit when the found ‘solutions’ do not ameliorate their well-being and continue exposing them to PD [10]. Thus, based on the drawn information, it is evident that PD correlates positively with employees’ ITQ.

Employees’ turnover intentions are often low when they adopt resilience as a coping strategy. Resilience is “the ability to preserve and rebound under conditions of stress and adversity” [15] (p. 4). Possessing such a capability can enhance one’s tolerance to various stressors. Ref. [16] holds similar perspectives, maintaining that resilience can reduce a person’s turnover intentions. However, [15,16] admit that attaining resilience is often difficult when the affected persons struggle with BU and PD. In their submission, [16] indicate that BU escalates when a person faces emotional exhaustion and depersonalization, lowering their accomplishment. In return, they become psychologically distressed and can hardly remain resilient. According to [15], heightened PD can expose one to anxiety and depression, making them inefficient, cynical, socially dysfunctional, emotionally exhausted, and with little confidence in executing the assigned role. Such happenings propel them to develop the urge to quit. Thus, drawing from this description, one can understand PD and BU as limiting people’s resilience and driving them toward the turnover intention.

Financial well-being (FWB) develops when individuals can meet their present and ongoing financial requirements. The capability enhances one’s feeling of being financially secure, improving their choice-making approaches aimed at enjoying life. While BU occurs for several reasons, studies have shown that individuals often feel emotionally exhausted when experiencing financial burdens [17]. The researchers assert that an organization’s move to ensure its employees’ financial security through improved wages can advance the latter’s well-being, lowering their turnover intentions. Ref. [18] concurs with [17], stating that increasing employees’ salaries boost their job satisfaction levels, lowering their emotional distress. According to [19], inadequate salaries and other limited financial rewards are often push factors for workers to leave for other companies offering better benefits. The four authors maintain that FWB is a motivational factor for employees. Hence, investing in FWB improves workers’ morale, diminishing their BU experiences and desire to quit.

A lack of financial security promotes employees’ feelings of job insecurity and can force them to quit their respective jobs. The COVID-19 pandemic caused financial anxiety among employees in the government and private sectors [20]. In their 2021 study, [20] highlighted that while the pandemic brought the issue into the light, workers often feel job-insecure when their organizations cannot assure them of their financial stability. Ref. [21] refers to it as financial self-efficacy, arguing that its perception can deepen one’s faith in attaining financial goals when working for an organization. When realized, financial self-efficacy can boost individuals’ emotional coping, helping them strive to embrace effective and functioning approaches to manage their pressure and continue working [21]. Such aspects depict FWB as an assuring approach to job security, lowering employees’ emotional exhaustion and turnover intentions.

BU and PD can push an employee to develop turnover intentions. Hotel industry employees, including men in the Middle East, face similar challenges, forcing them to quit. There is a gap in the existing literature about the moderating effects of FWB in the relationship between BU and ITQ, especially when it comes to male employees in the hotel industry in the Middle East. Sharjah, one of the seven emirates comprising the United Arab Emirates, was the location of the research. This emirate is less well-known than others, is rooted in Islamic culture, and has not been the focus of similar scholarly studies in the past.

The main objective of this research was to examine the influence of BU on male hotel employees’ ITQ, incorporating the mediating effect of PD and the moderating effect of FWB within the context of this particular population. Consequently, the purpose of this study was to evaluate whether ITQ and PD are outcomes of BU in this specific population sample. Determine the importance of PD in the relationship between BU and ITQ. In addition, it is required to examine if the FWB has any effects on the relations between the previously mentioned variables. Despite interventions such as supervisor support and good leadership styles, hotel workers continue to face elevated BU due to high workloads, tight deadlines, and variable shifts. Pandemics and greater competition also have an impact on the well-being of hotel personnel, and these happenings have lowered their morale, prompting them to develop the desire to leave. Besides the unfavorable work environment, hospitality workers struggle with anxiety and depression due to PD, increasing their desire to quit. It is essential to observe the many patterns of employee behavior at work in order to comprehend how they communicate their emotions [22], and this becomes yet more important in the hotel industry, which is a people-based industry [23] because hotel employees’ behavior directly impacts customer satisfaction [24].

The findings of this research show that BU significantly impacts ITQ and PD. PD partially mediates the BU and ITQ relationship, so this positive relationship is partially explained by the existence of PD. Although FWB is critical for workers’ motivation to perform and stay at their organizations, the current research found that FWB does not moderate the relation between BU and ITQ. Hotel organizations who assume that a pay rise or an annual bonus will erase all desires of their BU employees to quit their jobs are proven to be mistaken. FWB moderates the relationship between BU and PD, especially for those employees with higher financial prospects.

**Figure 1:**
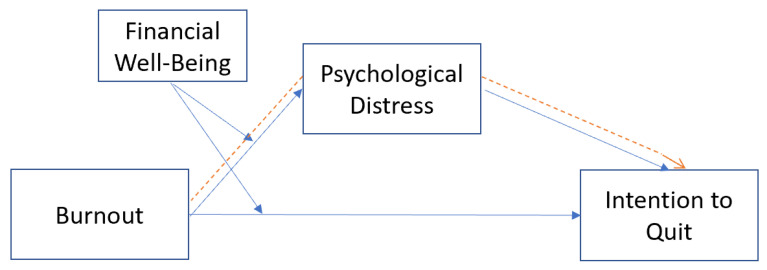
Conceptual framework.

Figure 1 displays the study’s conceptual framework.

## 2. Theoretical Background and Research Hypothesis

In BU research, the following are the predominant hypotheses pertaining to the determining variables: social cognitive theory [25], demands-resources theory [26], social exchange theory [27], organizational theory [28], and theory of emotional contagion [29].

The social cognitive theory emphasizes personal factors such as self-efficacy, self-confidence, and self-concepts as they relate to BU [30]. The job demands-resources model focuses on the idea that BU occurs when individuals are subjected to continuous job demands and have insufficient resources to address and minimize such needs [31]. According to the social exchange hypothesis, BU develops when a worker perceives an imbalance between their efforts and contributions and the outcomes of their employment [32]. According to organizational theory, BU is the result of organizational and job pressures paired with insufficient individual coping skills [33]. Emotional contagion is the tendency to reflexively imitate and synchronize facial expressions, vocalizations, postures, and movements with those of other individuals and, consequently, to emotionally converge with them [29].

The current research focuses on the outcomes of BU, both work and health-related. BU is believed to cause a deterioration in job performance and an increase in ITQ among employees. Furthermore, given that BU is a stress-related occurrence, it is expected that it will have major health repercussions for the individual and may also affect the individual’s personal life if they are experiencing PD [34].

In addition to the two BU outcomes identified by [34], the current study includes FWB. Financial circumstances are recognized as one of the most important determinants of an individual’s health. The rise in financial anxiety that results from a fall in FWB reduces a person’s health-related quality of life [35]. Ref. [36] research determined that, as an individual factor, FWB moderated the impact of COVID-19 anxiety and job insecurity on BU intensity.

### 2.1. Burnout and Its Relationship to Intention to Quit

Refs. [2,3,5] associate the continued BU and turnover intentions in the global hospitality industry with a lack of supervisor support. Ref. [3] argues that hotel employees’ successful mandate execution depends on their interactions with professional, environmental, and personal factors. However, with the growing competition in the hospitality industry, these workers face constant expectations to keep their performances high. As a result, [3] suggests the need for supervisor support, arguing that hospitality sector supervisors can help workers develop direct work approaches and lower their job overload. The four scholars also maintain that supervisor support enhances hotel workers’ emotional engagement through resilience and mindfulness, lowering their job BU and turnover intentions [3]. Refs. [2,5] hold similar views but decry inadequate executive support in the hotel industry, which they see as escalating employee exhaustion.

The hotel industry in the Middle East also grapples with employee BU, escalating turnover intentions. The region faced a tourism recession during the COVID-19 pandemic, which rendered its hospitality and service industry workers jobless. Despite such setbacks, [37] indicates that several hospitality companies in Middle Eastern countries have implemented recovery measures, helping them retain their employees and generate high profits. Unfortunately, they have not minimized workers’ BU, with the problem still prevalent in the United Arabs Emirates (UAE). For instance, in the UAE, 95% of hotel employees are expatriates with “different cultural backgrounds and variations in language, communication, and work skills” [38] (p. 39). Although the process has enhanced the employees’ abilities to deal with diverse customers, it has escalated job-related pressure, which, unfortunately, leads to BU and high turnover rates. The same problem extends to Saudi Arabia. In their 2019 study, which drew data from the Bureau of Labor Statistics, Ref. [39] determined that Saudi Arabia’s motel and hotel industry has a roughly 73.8% employee turnover rate, far higher than the recommended 10–15% range. The researchers decry poor working conditions comprising a lack of flexibility, inadequate work–life balance, rude employers with stern behaviors, less compensation, and insufficient promotion or appraisal [39]. These challenges emerge when there is still an expectation of hotel workers to deliver, propelling them to mental exhaustion and ITQ. Hence, this situation shows the Middle East’s hospitality/service industry as subjecting employees to BU, forcing them to develop the urge to leave.

Researchers have not established explicit findings on the gender most affected by BU, especially in the Middle East. Ref. [40] states that the global hotel industry’s workers are predominantly women. Such a large proportion has heightened the group’s exposure to high organizational commitment, with women struggling to maintain a healthy work–life balance, exposing them to BU. Although [41] raises similar issues, they maintain that the hotel industry’s upper management echelons are still male-dominated. Despite the fact that the situation is changing worldwide, “it remains more entrenched in Middle Eastern countries” [41] (p. 573). Ref. [42] concurs with this idea. Women constitute approximately 50–70% of the hotel workforce globally. Unfortunately, the Middle East’s gender composition is still male-dominated, with women accounting for only 22.3 and 40.9% of hotel employees in the Kingdom of Saudi Arabia (KSA) and UAE, respectively [42]. Such male dominance leaves men with extreme expectations to fulfill in their work environments, leading to extreme BU. Therefore, although there is a gap in this discourse, one can deduce that male workers in the Middle East’s hotel industry are more affected by BU than female employees since they constitute the majority.

**H1.**  *Burnout has a significant relationship with the intention to quit*.

### 2.2. Burnout and Its Relationship to Psychological Distress

The hotel industry is not free from BU and PD. The problem relates to workplace demand, as hospitality workers are often “required to perform various unrelated tasks in a very limited timeframe and without adequate training” [43] (p. 2). The hotel environment is unfavorable due to its unrealistic deadlines and unpredictable shiftwork, taxing employees’ emotional and physical capabilities. Such issues increase work-related stress. Although workplace demands also have emotional connotations, [44] note that hotel environments have failed to improve workers’ work–life quality, subjecting them to emotional exhaustion, depersonalization, and limited personal accomplishment. Ref. [45] agrees with the latter’s assertions, stating that the hospitality workplace’s stressful and prolonged demands can negatively affect employees’ psychological states.

The prolonged BU in the hospitality industry also draws from the incivility of customers and supervisors. Ref. [46] discusses how the hotel industry deals with multiple types of customers, with some treating workers in uncivil manners. While such acts might not include unfair behaviors, their long-term impacts can be offensive and intense, with employees becoming emotionally exhausted. Overcoming this problem relies mainly on supervisor support. Unfortunately, the hospitality industry has not invested in training its supervisors to tackle such behavioral issues to create a favorable working environment [46]. The failure to eliminate incivility leads to workplace loneliness. In their view, Ref. [47] highlights that the problem often prompts hotel employees to desire psychological detachment, especially from their mandates. However, since these workers operate under tight schedules and with extreme workloads, they can barely overcome the stated challenges. As a result, they find themselves exposed to BU, which escalates to PD.

**H2.**  *Burnout has a significant relationship with psychological distress*.

### 2.3. Psychological Distress and Its Relationship to Intention to Quit

Hospitality industry workers face PD due to prolonged aggressive leadership, which augments turnover intentions. Ref. [48] points out that the adopted leadership approaches can determine service delivery in the hotel industry, urging the involved organizations to maintain quality relationships between employees and their leaders, specifically supervisors. Leaders should uphold understanding, transformational, empowering, and authentic leadership since they have positive “effects on hospitality employees” [48] (p. 3565). Ref. [49] holds similar views, insisting that hotel industry leaders can become effective when they uphold laissez-faire, transactional, and transformational leadership styles. They regard the latter as effective in lowering employees’ turnover intentions. In relation to these suggestions, [48] decry the prevailing despotic leadership, which has created the space for aggressive behaviors toward hotel workers. Besides heightened exploitation, this leadership style has made the hospitality environment unfavorable for employees, creating stress and fear among them regarding their positions and roles in their respective organizations [48].

The hotel industry environment is highly unfavorable, creating the space for increased employee stress. Besides the heavy workloads and irregular schedules hospitality employees grapple with, the industry employs workers temporarily with qualifications that fail to meet the set job requirements [50]. Such instances increase stress, especially when the hired workers cannot meet their mandates’ expectations. The prolonged existence of these expectations only degrades hotel laborers’ well-being, exposing the latter to PD [51]. While some cope, others become overwhelmed with work demands, thus exceeding their coping abilities [50]. Although [52] suggests employee training to improve their job awareness and lower the consequences of workplace ostracism, [50] maintains that realizing such changes is often difficult when the affected group already feels overwhelmed with their responsibilities. As a result, these employees develop turnover intentions, viewing quitting as the only solution to the experienced PD. Thus, based on this review, we can see that the hotel industry promotes an unfavorable working environment, distressing workers and subjecting them to high turnover intentions.

**H3.**  *Psychological distress has a significant relationship with the intention to quit*.

### 2.4. The Role of Psychological Distress in Relation to Burnout and Intention to Quit

Developing ITQ also relates to the limited social support the affected persons receive. Ref. [53] acknowledges poor social support, particularly in the workplace, as causing extreme emotional exhaustion and distress. The three researchers mainly focus on supervisor support, noting that the latter affects employees’ confidence more than psychological demand. When an organization fails to provide the needed social support to its workers, it deprives them of reciprocity, leading to the depletion of the group’s emotional resources [53]. Ref. [54] argues that support provision enhances workers’ adaptation to job-related stressors, diminishing their turnover intentions. Although [55] have paid attention to peer support, they agree that its inadequacy augments BU, negatively affecting one’s work execution. These aspects expose the impacted workers to depression and anxiety, with many viewing quitting their positions as the best solution to reducing their PD [56]. Therefore, individuals’ turnover intentions increase with prolonged BU and PD.

Scholars have not exempted turnover intentions in the hospitality/service industry due to PD and BU. Refs. [57,58] indicate that the hotel industry faces continued labor shortages due to high turnover rates. These have forced the organizations’ management to shift the workload to the few remaining workers, straining them psychologically. Offered limited organizational support, these service employees struggle to meet customer demands and maintain a healthy work–life balance [59].

Workplace bullying in the hospitality/service industry has also led to employee BU and PD, raising turnover intentions. Ref. [60] cites workplace bullying as a significant problem the hospitality industry faces, damaging workers’ capacity for cooperation and the organizational environment. Researchers report that roughly 16% of hospitality employees have become subjected to undesirable acts in the workplace [60]. Although [61] suggests that organizational commitment effectively boosts employees’ morale and limits their BU, [60] insists that the propagated bullying often hinders workers’ adoption of any coping strategy. In return, the bullying-related pressure and stress increase the employees’ exhaustion, negatively affecting the quality of their service delivery. With prolonged bullying, workers struggle to cope with the experienced PD, forcing them to develop turnover intentions.

**H4.**  *Psychological distress mediates the relationship between burnout and the intention to quit*.

### 2.5. The Role of Financial Well-Being in Relation to Burnout and Intention to Quit

Scholars consider FWB a major concern for lowering turnover intentions in the hospitality/service industry. Ref. [62] states that, despite many hotel workers’ temporary employment, hoteliers only focus on refining worker services to achieve sustainable competitive advantages. Such an approach involves improving employees’ job satisfaction, which the researchers insist relates to their low intent to quit [62]. Despite the importance of these processes, hospitality workers continue to complain about their low pay [63]. Although the current organizational leaders are looking into employee turnover in the hospitality industry, they have sidelined workers’ monetary rewards, with the group still recording low tangible benefits and salaries [63].

Another challenge in attaining FWB in the hospitality industry involves hiring relatively low-skilled workers to deliver high-quality services in a rapidly transforming environment. The “need for a large number of relatively low-skilled employees may serve to depress earnings if wages need to be controlled to ensure profitability” [64] (p. 2). While comparing personnel earnings across industries, [64] noted that hospitality industry employees receive below-average salaries, leading to a struggle to maintain financial stability. Ref. [36] regards such assertions as valid, adding that when a company encourages high FWB for its laborers, it makes them confident of remaining resistant to any economic shock that may arise. However, since the hotel industry prefers low-skilled workers, it fails to provide adequate remuneration, causing workers to struggle with balancing their needs with the meager payments they receive. Such happenings lead to BU and high turnover intentions [62]. Drawing from this review, it remains evident that the hospitality industry’s lack of FWB and high workload expose employees to BU and increase their intention to quit.

**H5.**  *The impact of burnout on employees’ intention to quit varies depending on their financial well-being*.

### 2.6. The Role of Financial Well-Being in Relation to Burnout and Psychological Distress

FWB appears to lower BU, which, in turn, decreases PD. Ref. [65] argues that employee efforts depend on job-related benefits, including financial gains. The authors indicate that adverse job characteristics, such as low pay, can attract BU, negatively affecting their service delivery and lives, with some developing psychosomatic and mental disorders [65]. Accompanied by the absence of job control and the heightened workload, employees with poor remuneration may feel overwhelmed with the set expectations, prompting BU and stress [66]. Organizational managers should provide non-monetary rewards to their employees since “monetary rewards alone cannot decrease the stressful characteristics of one’s job” [67] (p. 190). Although the assertion is valid, [68] disagree, positing that workers’ choice to work for a specific company correlates with the offered financial benefits. These scholars maintain that most employees desire an effort–reward ratio, with the provided financial gains aligning with their efforts [68]. Such expectations extend to the non-profit sector. According to [69], job demands in this sector also depend on monetary gains. Despite the contrasting opinions on how to approach the issue, the analyzed scholarly sources agree that focusing on employees’ FWB can motivate them to work, reducing their feelings of BU and PD.

The mandate to reduce hospitality workers’ BU and PD levels through FWB fall on organizational managers, who are the leaders. Ref. [70] view hotel managers as crucial in designing the best financial rewards to improve employees’ attitudes toward their mandates. On the other hand, [71] suggests the need to adopt a transactional leadership style, where leaders set worker-friendly expectations and specify the associated rewards. These mechanisms can increase employees’ productivity since they would not feel sidelined in the organization. The process lowers the workers’ likelihood of experiencing BU, which also reduces their PD.

**H6.**  *The impact of burnout on employees’ psychological distress varies depending on their financial well-being*.

## 3. Materials and Methods

### 3.1. Participants and Procedure

Between 1 September and 30 September 2022, data were collected from five hotels located in Sharjah, United Arab Emirates. The hotels were rated four and five stars, with some being strictly business hotels and others being resorts. Three hotels were handled by three distinct global hotel chains, one of which was franchised, and the other one was privately managed. The five General Managers were contacted, the research objectives, including employee and hotel anonymity, were described, and permission was gained.

A designated individual coordinated with each hotel’s General Manager and Human Resources Manager to distribute 400 questionnaires. Through the aforementioned GMs, HR management communication and approval were obtained. The questionnaire’s respondents received no reward. The response rate was 76%, as 304 questionnaires were returned as valid samples. Male employees who worked directly for the hotel, for an outsourced company providing temporary services (casual personnel in housekeeping, restaurants, etc.), or for an external company operating a business in the hotel constituted the sample.

The questionnaire included 24 Likert (1–5) scale items (see Appendix A) and five sociodemographic profile questions (see Table 1). BU, ITQ, PD, and FWB were evaluated. The questionnaire was approved by a team of specialists composed of academics from European and UAE universities (3) and hospitality experts (3). The number of expert opinions taken for this research exceeds the minimum of two required to confirm the measurement scale [72]. The experts checked for grammatical errors and considered how respondents would view the questions’ original context and intended audience. Additionally, a pre-test was conducted with 40 Bachelor of Business Management students from the UAE to ensure the questionnaire was easy to understand. Academics were assisted by hospitality professionals in understanding the reality of the composition of hotel staff in the United Arab Emirates, and university academics assisted hotel professionals in understanding the importance of a rigorous methodology when developing a questionnaire using constructs from previous studies. The panel proposed minor textual modifications and recommended maintaining the original number of entries.

Partial least squares (PLS) were used to evaluate the proposed model and related hypothesis tests, which was sufficient for the number of respondents (*N* = 304).

### 3.2. Survey Instruments

BU was measured through a 7-item scale adapted from [73]. A sample item is “I feel burnt out because of my job.” This scale’s Cronbach alpha (reliability measure) is 0.85. ITQ was measured through a 7-item scale adapted from [25,74], and a sample item is “I often think about quitting my job.” The reliability for this scale was 0.83. PD was measured through a 7-item scale adapted from [75], and a sample item is “I feel ill at ease with myself.” The Cronbach’s alpha of this scale was 0.85. FWB was measured through a 3-item scale adapted from [76], and a sample item is “I pay my bills on time.” The Cronbach’s alpha was 0.85.

### 3.3. Common Method Bias

When data on exogenous and endogenous constructs are gathered from the same source simultaneously, common method bias (CMB) may emerge. Because we also obtained cross-sectional data, it is prudent to examine CMB [77,78]. We used Harman’s single factor to check for potential CMB concerns, and the variance had to be lower than 50% [79]. According to Harman’s single-factor findings, the first component explains 31.475% of the total variation under the 50% cutoff (see Appendix B). This verifies that the dataset used for this research does not contain CMB.

## 4. Data Analysis and Results

Structural equation modeling (SEM) is one of the most prominent research tools in a wide range of fields, including hospitality management. Recent research recommends the use of partial least square–structural equation modeling (PLS-SEM) as an appealing alternative to covariance-based structural equation modeling (SEM) [80]. For evaluating the presented hypotheses, partial least square–structural equation modeling (PLS-SEM) via SmartPLS 4 was used [81]. PLS-SEM is extensively used in management sciences and behavioral research [25,82,83,84] and is excellent for simple and complex models [85].

### 4.1. Measurement Model

**Figure 2:**
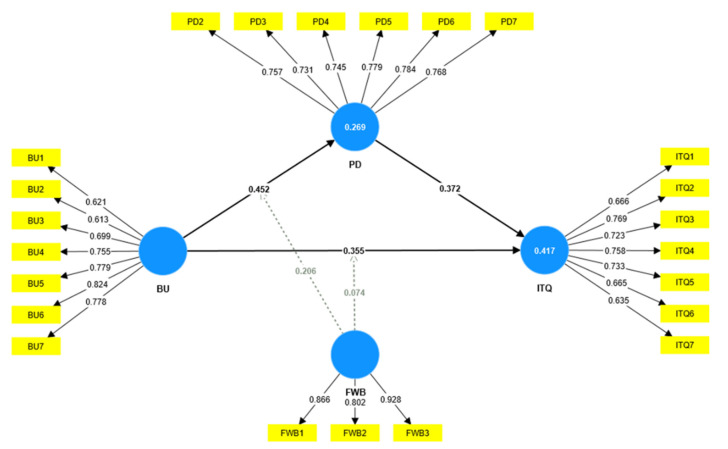
Measurement model.

To assess the validity and reliability of scales, PLS-SEM provides several tests. For instance, factor loading may be used to determine the reliability of each distinct indicator. According to [85], the minimum item loading should be greater than 0.5. Except for PD1, which had a loading below the minimum value of 0.5 and was thus eliminated, all of the individual items had loading values between 0.613 and 0.928 (see Figure 2). Similarly, Cronbach’s alpha and composite reliability (CR) were used to assess the internal consistency of scales that had been modified. The α and CR values should be more than 0.7, as suggested by [86]. Table 2 shows that all scales had α and CR values above 0.7. Additionally, the convergent validity was verified using the average variance extracted (AVE). AVE values of 0.5 or above are advocated for sufficient convergent validity [87]. As shown in Table 2, all the measures had AVE values greater than 0.5, thus offering convergent validity at a sufficient level.

In addition, discriminant validity was confirmed using the conventional [87] criteria and the contemporary Heterotrait–Monotrait Ratio (HTMT) criterion [81], as well as cross-loadings [85]. The first view argues that the diagonal value should be bigger than all other values in that row and column, while the latter premise proposes that all correlation values should be less than 0.8 [81]. The findings in Table 3 validate the discriminant validity of the model using both of these well-known techniques. In addition, researchers investigated cross-loading to give more evidence in establishing the study’s discriminant validity. The findings demonstrate that all of the data met the criterion. All required indicators have significant loadings (>0.6) on their indicated variables but modest loadings on other factors. This also supports the model’s discriminant validity. Table 4 presents the discriminant validity results.

PLS-SEM also allows for determining the effect size (*F*^2^), multicollinearity (VIF), determination coefficient (*R*^2^), predictive relevance (*Q*^2^), and model fit. The coefficient of determination reveals the variation in endogenous variables due to exogenous factors. The effect size demonstrates how the elimination of an external variable influences an endogenous variable. Regarding *F*^2^, we followed the recommendations of [88], according to which *F*^2^ > 0.02, 0.15, and 0.35 represent small, moderate, and large effect sizes, respectively. The *F*^2^ effect sizes of the variables in the current research range from low to large, confirming the robustness of the model. According to the views of experts, if the inner VIF values are <5, there are no collinearity concerns regarding the data [85]. According to the findings of the current investigation, the inner VIF values of measures range between 1.024 and 1.368. This demonstrates there is no collinearity problem in the present study’s data and validates the model’s strength. There is no formal rule about *R*^2^ levels, but as a guideline, values of 0.25, 0.50, and 0.75 are considered low, moderate, and substantial, respectively [85]. According to Table 5, both outputs were within the moderate range. Predictive relevance must exceed zero. As a rule, *Q*^2^ values may be classified as having significant (>0.50), medium (>0.25), or small (>0) predictive relevance for the model [86]. Table 5 demonstrates that all *Q*^2^ values were greater than zero and were within the moderate range of predictive relevance. Standardized root means squared residual (SRMR) was used to validate the model. An SRMR value less than 0.08 indicates the fitness of the model [89]. Our model’s SRMR score of 0.074 satisfies the requirements for model fitness.

### 4.2. Hypothesis Testing

**Figure 3:**
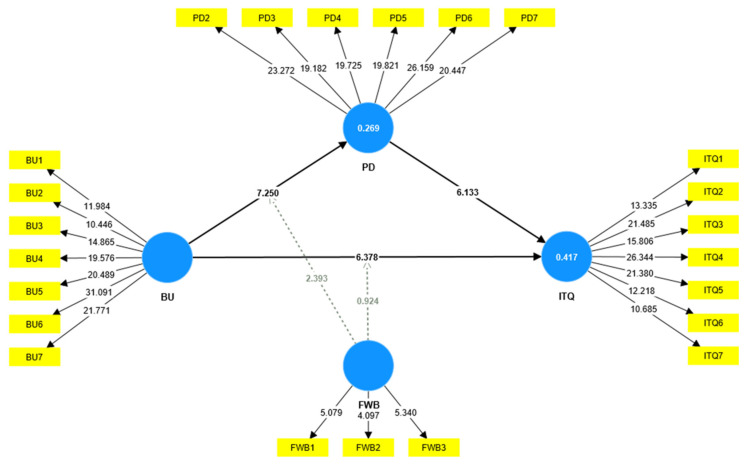
Structural model.

With a satisfactory measurement model assessment approach, the bootstrap resampling technique with 5000 resamples was employed to evaluate the significance of the proposed hypotheses [90]. Table 6 and Table 7 and Figure 3 illustrate the results of direct, indirect, and moderating hypotheses. The *p*-value and *t*-value are used to test hypotheses. Hypotheses are accepted if the *p*-value is less than 0.05, the *t*-value is above 1.96, and vice versa. First, the results (see Table 6) of direct relationships demonstrate that BU positively links to ITQ (β = 0.355, *t*-value = 6.378, *p* < 0.001) and PD (β = 0.452, *t*-value = 7.250, *p* < 0.001). In addition, PD positively links to ITQ (β = 0.372, *t*-value = 6.133, *p* < 0.001). This thus supports the three direct hypotheses of H1, H2, and H3.

Second, the mediation effect of PD was examined in the relationship between BU and ITQ. The results indicate that PD significantly mediates the relationship between BU and ITQ (β = 0.168, *t*-value = 4.850, *p* < 0.001). Moreover, based on significant direct, indirect, and total effects, it can be concluded that PD partially mediates the relationship between BU and ITQ. Hence, H4 was supported. The results of the mediation analysis are presented in Table 7.

**Figure 4:**
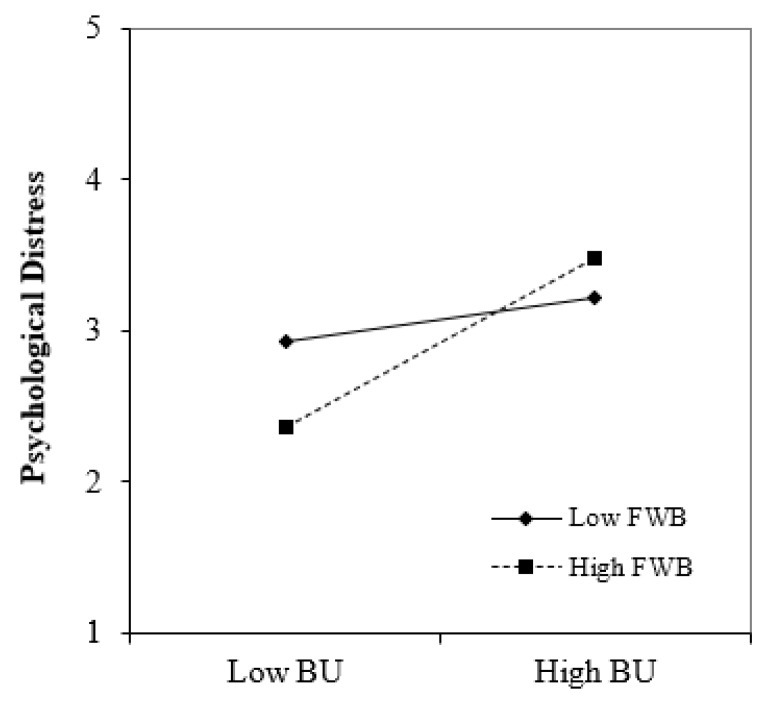
Effect of burnout and financial well-being on psychological distress.

Finally, the moderation effects of FWB were checked using the product indicator approach. The PLS-SEM results in Table 6 depict that FWB does not moderate BU and employees’ ITQ relationship (β = 0.074, *t*-value = 0.924, *p* > 0.05). Therefore, H5 was not supported. Moreover, hypothesis H6 tested the moderation effect of FWB. The results in Table 6 indicate that FWB positively moderates the BU and PD relationship (β = 0.206, *t*-value = 2.393, *p* < 0.001), as shown in Figure 4. This indicates FWB makes the following relationship stronger.

Moreover, the contextual analysis revealed an insignificant relationship between contractual relationship and ITQ; however, a significant negative relationship was found amongst position, experience, and ITQ.

Finally, researchers have suggested a novel calculating method for the study model’s predictive relevance, which was created mainly for the PLS prediction-oriented character of SEM [91]. Additionally, it is necessary to first determine the *Q*^2^ of the LVs, and if the *Q*^2^ value is larger than zero, then we can determine the items [91]. When the value of the PLS-LM of all items is higher, it indicates no predictive power; when the value of the PLS-LM of all items is less, it indicates stronger or higher predictive ability, and when the majority of items have lower PLS-LM values, it indicates medium predictive power [91]. Table 8 shows that most items had lower PLS-LM scores, suggesting medium predictive power, with *Q*^2^-predict being larger than zero.

## 5. Discussion and Conclusions

The purpose of this research was to deepen the understanding of workplace behavior concepts affecting hotel employees today (BU, ITQ, PD, and FWB) and to propose a model that shows their relationship and impact on managerial implications.

This research empirically studied the effect of BU on ITQ among male hotel employees, integrating the mediating effect of PD and the moderating effect of FWB. This was assessed through 304 surveys completed in five four- and five-star hotels in the UAE in September 2022. This research used the SmartPLS 4 software package to test hypotheses in a moderation mediation model.

The following is a summary of the findings.

First, all of the direct links in the model were positive and significant. Ref. [3] posits that although hospitality organizations fear losing their already established human capital, very few have invested in supervisory support to assist their employees in overcoming BU and lower their willingness to quit. The hotel industry workers’ prevailing BU and turnover intentions have been intensified by limited supervisor support. Despite suggesting the need to uphold mindfulness to inhibit the emotional exhaustion experienced among hotel employees, [45] admits that the extreme demands make the hospitality space unaccommodating to emotional stability. These instances can upscale BU among hotel workers, which, unfortunately, pushes them into PD. Working in the hotel industry alone is straining due to the intense “physical labor, tight schedules, long hours of work, irregular shifts, and recurring tasks” [51] (p. 139). When combined with despotic leadership, these strains can cause PD among hotel workers, with many developing the urge to quit.

Second, PD was found to partially mediate the relationship between BU and ITQ. Despite the push to implement Employee Assistance Programs (EAPs) across hospitality enterprises, [59] admit that the intense pressure due to workload has continued to limit employees’ emotional and psychological stability. Since such strains cause BU and PD, they escalate turnover intentions, exposing the hospitality industry to further labor shortage threats.

Third, it was found that FWB does not moderate the BU and ITQ relationship. The positive relationship between BU and ITQ does not change regardless of the financial well-being of the employee. These research findings are contrary to [92], who reported that providing financial rewards to hotel employees can motivate them to continue working. This is also contrary to research statements such as those in [93], maintaining that the hotel industry still struggles with poor remuneration, causing a direct decline in workers’ job satisfaction and making FWB an important factor influencing BU and its consequences. Since the hospitality sector also overloads its laborers, many end up feeling exhausted through BU, dissuading them from continuing to work in the affected organizations. Accompanied by low pay, these workers struggle to keep up with company expectations, prompting them to develop their intention to leave.

Fourth, it was found that FWB does moderate the relationship between BU and PD, especially for those employees with higher financial prospects. Overcoming BU and PD in the hospitality/service industry requires investing in employees’ FWB. Ref. [94] revealed that BU is highly prevalent among hospitality employees due to the intense workloads and low pay. Although they agree that the hospitality industry operates on seasonality, [95] urges the involved stakeholders to reward their workers. The scholars maintain that this mechanism can help employees adopt the organization’s values and feel comfortable when executing their roles. Ref. [94] also decries the reward–effort imbalance in the hotel industry, leaving employees feeling as if they are doing their organizations a favor by working for them. The process also lowers BU and money-related PD.

Fifth, it was found that the contractual relationship in the organization has an insignificant relationship with ITQ. On the other side, position (lower to top management) and experience (years in the work) revealed a significant negative relationship with ITQ.

On a theoretical level, this study expands upon [34]’s findings regarding the effects of burnout in the workplace (ITQ) and the personal environment (PD). It adds the insight that PD acts as a partial mediator between BU and ITQ. FWB is only included as a moderator in select instances of the relationships between the aforementioned variables.

Consequently, the implications of the results at all levels (hotel business management, employee and customer satisfaction, and human resources) are examined in the next paragraph.

Despite control variables not being the main aim of the research, it was found that contractual relationship in the organization has an insignificant relationship with ITQ; further, position (lower to top management) and experience (years in the work) revealed a significant negative relationship with ITQ. The contractual relationship in the UAE does not have strong implications for employees since their contract can be ended at any time by the employer paying an end-of-service fee. Therefore, if an employee has the intention to leave, being on a permanent or outsourced contract becomes irrelevant. On the other hand, the position and years of experience that an employee has in these hotels were revealed to negatively impact their intentions about leaving their job; therefore, employees with higher positions are less likely to have the intention to quit, and employees with more years of experience are also less likely to have the intention to quit.

The most important management implication of this study for organizations is that a more careful approach should be taken toward employees’ state of BU, particularly when it is visible that BU is impacting employees’ PD and ITQ. The effects of burnout are costly not just for the employee and his or her personal life but also for the workplace.

This research has proven that PD partially mediates the positive relationship between BU and ITQ, so the approach to PD should also be considered, this being an important factor in the BU–ITQ relationship.

Hotel employees’ job satisfaction is affected by the way managers lead their teams due to the special factor of human relationships in hospitality [96]. Many other factors can also affect hospitality employees’ job satisfaction. Employees who are experiencing BU are a burden to their coworkers in a number of ways, including increasing the likelihood of interpersonal conflict and creating more work delays. Accordingly, BU can be contagious and spread through interpersonal contacts at work [97,98]. In order to prevent the spread of BU, hotel HR managers should focus their attention not just on employees who have been identified as suffering from BU but also on their peers. HR should have routine meetings with employees in order to spot BU situations and intervene before they spread to other employees. To avoid highlighting and magnifying BU’s condition, it would be prudent to assess this issue concurrently with ordinary performance evaluations.

Employees exhibit undesirable behaviors when they feel BU in their current job and having a good FWB does not moderate their ITQ. This is a significant problem in the hotel business because most business organizations and managers think that a pay rise or an annual bonus can eliminate employees’ ITQ without paying attention to the actual issues that are generating the state of BU. The focus should be placed on the reasons why employees are experiencing BU.

FWB has proven to moderate employees’ PD, especially for those employees with higher financial prospects. Employees with higher FWB seem to be more sensible when experiencing BU, so PD is higher for them. This finding can help HR managers in hotels to focus on these employees experiencing BU and develop a special approach to the PD they might be experiencing.

Therefore, a new approach toward FWB should be taken by hotel organizations and HR managers when evaluating the employee climate, and they should focus on non-monetary rewards as an option to influence this issue.

Despite its achievements, this research has limitations that could be addressed by future research. First, all five sample hotels were located in Sharjah, United Arab Emirates—the same emirate and country. Future research could examine hotels in various countries. Second, the questions for this cross-sectional study were developed during the month of September 2022. As a longitudinal study, carrying out surveys at different times could add value to this research. Thirdly, the model examined the effect of PD as a mediator. Including evaluations of additional mediating effects, such as the comfort zone, when applicable, would be advisable when seeking to acquire a deeper knowledge of this subject. Fourthly, the use of self-report scales as the sole method of data collection could compromise the veracity of the gathered information. Future research could attain greater rigor and informative depth by combining additional approaches like in-depth interviews. Finally, this study focuses primarily on the male population, but similar studies on the female population will also contribute to the current level of research in this field.

## Figures and Tables

**Table 1:** Demographics.

Category	Sub-Category	Frequency	Percent
Contractual relationship			
	Permanent	164	54.0
	Outsourced	140	46.0
	Total	304	100.0
Age			
	18–25 years old	37	12.2
	26–35 years old	113	37.2
	36–45 years old	103	34.3
	>45 years old	51	16.3
	Total	304	100.0
Position			
	Lower management	151	49.7
	Middle management	122	40.1
	Top management	31	10.2
	Total	304	100.0
Experience			
	Less than a year	53	17.4
	1–3 years	82	26.9
	4–6 years	88	28.8
	>6 years	81	26.6
	Total	304	100.0
Education			
	Primary school	99	32.6
	High school	100	32.9
	University or above	105	34.5
Total		304	100.0

**Table 2:** Measurement model results.

Constructs	Items	Loadings	VIF	CA	CR	AVE
Burnout	BU1	0.621	1.526	0.851	0.887	0.503
	BU2	0.613	1.576			
	BU3	0.699	1.659			
	BU4	0.755	1.817			
	BU5	0.779	2.200			
	BU6	0.824	2.303			
	BU7	0.778	1.837			
Intention to Quit				0.834	0.875	0.502
	ITQ1	0.666	1.576			
	ITQ2	0.769	2.074			
	ITQ3	0.723	1.780			
	ITQ4	0.758	2.525			
	ITQ5	0.733	2.271			
	ITQ6	0.665	1.779			
	ITQ7	0.635	1.696			
Psychological Distress				0.854	0.892	0.579
	PD1	-	-			
	PD2	0.757	1.664			
	PD3	0.731	1.661			
	PD4	0.745	1.697			
	PD5	0.779	1.981			
	PD6	0.784	1.830			
	PD7	0.768	1.857			
Financial Well-being				0.845	0.900	0.751
	FWB1	0.866	2.364			
	FWB2	0.802	1.883			
	FWB3	0.928	2.018			

Note: VIF, variance inflation factors; CA, Cronbach’s alpha; CR, composite reliability; AVE, average variance extracted.

**Table 3:** Discriminant validity.

	Heterotrait–Monotrait Ratio				Fornell–Larcker Criterion			
Constructs	BU	FWB	ITQ	PD	BU	FWB	ITQ	PD
BU					0.728			
FWB	0.069				−0.003	0.867		
ITQ	0.624	0.077			0.548	0.016	0.709	
PD	0.559	0.068	0.657		0.486	−0.051	0.559	0.761

**Table 4:** Cross-loadings.

Items	BU	FWB	ITQ	PD
BU1	0.621	−0.015	0.345	0.361
BU2	0.613	−0.067	0.213	0.246
BU3	0.699	−0.080	0.369	0.308
BU4	0.755	0.029	0.419	0.354
BU5	0.779	0.029	0.384	0.371
BU6	0.824	0.040	0.512	0.394
BU7	0.778	0.006	0.465	0.407
FWB1	0.015	0.866	0.041	−0.021
FWB2	−0.033	0.802	−0.008	−0.035
FWB3	0.004	0.928	0.012	−0.062
ITQ1	0.488	−0.049	0.666	0.369
ITQ2	0.394	−0.003	0.769	0.423
ITQ3	0.297	0.107	0.723	0.328
ITQ4	0.447	0.027	0.758	0.404
ITQ5	0.393	−0.041	0.733	0.354
ITQ6	0.340	0.016	0.665	0.440
ITQ7	0.318	0.044	0.635	0.435
PD2	0.388	0.010	0.484	0.757
PD3	0.328	−0.045	0.408	0.731
PD4	0.329	−0.090	0.409	0.745
PD5	0.333	−0.026	0.415	0.779
PD6	0.436	0.001	0.423	0.784
PD7	0.391	−0.093	0.407	0.768

**Table 5:** Effect size, coefficient of determination, and blindfolding results.

	*F* ^2^		VIF		*R* ^2^	SSO	SSE	*Q*^2^ (= 1 − SSE/SSO)
	ITQ	PD	ITQ	PD	Endogenous Constructs			
BU	0.164	0.268	1.318	1.039				
FWB	0.001	0.008	1.032	1.024				
ITQ					0.417	2128.000	1712.123	0.195
PD	0.173		1.368		0.269	1824.000	1549.783	0.150

Note: SSO, sum of squares of observations; SSE, sum of squares of predictions errors.

**Table 6:** Direct and moderating effects.

Hypotheses	Relationships			Path Coefficient	STDEV	BCI-LL, BCI-UL	T-Statistics	*p*-Values	Conclusion
	IV	M	DV						
Direct Effects									
H1	BU	→	ITQ	0.355	0.056	0.248, 0.469	6.378	0.000	Significant
H2	BU	→	PD	0.452	0.062	0.331, 0.575	7.250	0.000	Significant
H3	PD	→	ITQ	0.372	0.061	0.251, 0.490	6.133	0.000	Significant
Moderating Effects									
H5	FWB × BU	→	ITQ	0.074	0.080	−0.118, 0.193	0.924	0.356	Insignificant
H6	FWB × BU	→	PD	0.206	0.086	−0.014, 0.325	2.393	0.017	Significant
Control Effects									
Contractual relationship		→	ITQ	0.111	0.114	−0.113, 0.335	0.974	0.331	Insignificant
Position		→	ITQ	−0.220	0.085	−0.388, −0.052	−2.583	0.010	Significant
Experience		→	ITQ	−0.148	0.054	−0.253, −0.042	−2.755	0.006	Significant

Note: IV, independent variable; DV, dependent variable; STDEV, standard deviation; BCI, Bias Confident Interval.

**Table 7:** Direct, indirect, and total effects.

	Direct Effects			Indirect Effect			Total Effects			
Hypothesis 4	β	T Statistics	*p*-Values	β	T Statistics	*p*-Values	β	T Statistics	*p*-Values	Conclusion
BU→PD→ITQ	0.355	6.378	0.000	0.168	4.850	0.000	0.523	10.558	0.000	Partial mediation
BCI-LL	0.248			0.107			0.427			
BCI-UL	0.469			0.244			0.622			

**Table 8:** PLS-predict.

	PLS		LM		PLS-LM		Q^2^ Predict
	RMSE	MAE	RMSE	MAE	RMSE	MAE	
ITQ1	0.741	0.594	0.755	0.601	−0.014	−0.007	0.228
ITQ2	0.750	0.602	0.760	0.608	−0.010	−0.006	0.145
ITQ3	0.853	0.645	0.854	0.647	−0.001	−0.002	0.061
ITQ4	0.882	0.660	0.880	0.651	0.002	0.008	0.188
ITQ5	0.886	0.683	0.892	0.681	−0.006	0.002	0.137
ITQ6	0.796	0.617	0.807	0.625	−0.011	−0.008	0.096
ITQ7	0.896	0.671	0.880	0.676	0.016	−0.005	0.087

## Data Availability

The data presented in this study are available on request from the corresponding author.

## Appendix A

**Table A1:** Survey instrument.

Constructs	Items
Burnout	
	I often feel physically exhausted on my job.
	I feel burnt out because of my job.
	I feel emotionally exhausted at work.
	I feel like every working hour is tiring for me.
	Working in direct relation with customers often drains my energy at work.
	I am tired of working directly with customers.
	I sometimes wonder how long I will be able to continue working with customers.
Intention to Quit	
	I often think about quitting my job.
	There is an excessive workload and time pressure at my workplace.
	There are no prospects for growth and promotion at work.
	I get paid less than I work for my company.
	There are no or poor training and development prospects in my company.
	I experience stagnation in growth by working in my current job due to the lack of opportunities.
	I keep on finding reasons to quit my current job.
Psychological Distress	
	I am currently avoiding others as much as possible.
	I have difficulties facing my problems.
	Lately I have no patience.
	I am currently aggressive about everything and nothing.
	I fell ill at ease with myself.
	I feel like throwing everything to the wind, quitting.
	I am now less receptive to the ideas and opinions of others.
Financial Well-being	
	I pay my bills on time.
	I have no concerns about covering my regular living expenditures.
	I am not currently in debt.

## Appendix B

**Table A2:** Harman’s single factor test.

	Total Variance Explained					
Component	Initial Eigenvalues			Extraction Sums of Squared Loadings		
	Total	% of Variance	Cumulative%	Total	% of Variance	Cumulative%
1	7.554	31.475	31.475	7.554	31.475	31.475
2	2.381	9.923	41.398			
3	2.019	8.412	49.810			
4	1.713	7.136	56.946			
5	1.146	4.777	61.723			
6	1.065	4.437	66.161			
7	0.940	3.916	70.076			
8	0.765	3.187	73.263			
9	0.686	2.858	76.122			
10	0.597	2.486	78.608			
11	0.569	2.371	80.979			
12	0.520	2.166	83.144			
13	0.455	1.897	85.042			
14	0.435	1.814	86.856			
15	0.428	1.784	88.640			
16	0.414	1.724	90.364			
17	0.401	1.672	92.035			
18	0.337	1.404	93.439			
19	0.316	1.318	94.757			
20	0.295	1.230	95.987			
21	0.273	1.139	97.126			
22	0.262	1.093	98.219			
23	0.225	0.938	99.157			
24	0.202	0.843	100.000			
